# Supplementary material for: Autosomal genetic variation is associated with DNA methylation in regions variably escaping X-chromosome inactivation
Source: Nat Commun. 2018 Sep 14;9:3738. doi: 10.1038/s41467-018-05714-3 (PMC6138682; doi:10.1038/s41467-018-05714-3)
Supplement: Supplementary file 13 — Supplementary Information [file 41467_2018_5714_MOESM13_ESM.pdf]

Autosomal genetic variation is associated with DNA methylation in regions variably escaping X-chromosome inactivation

Luijk *et al.*

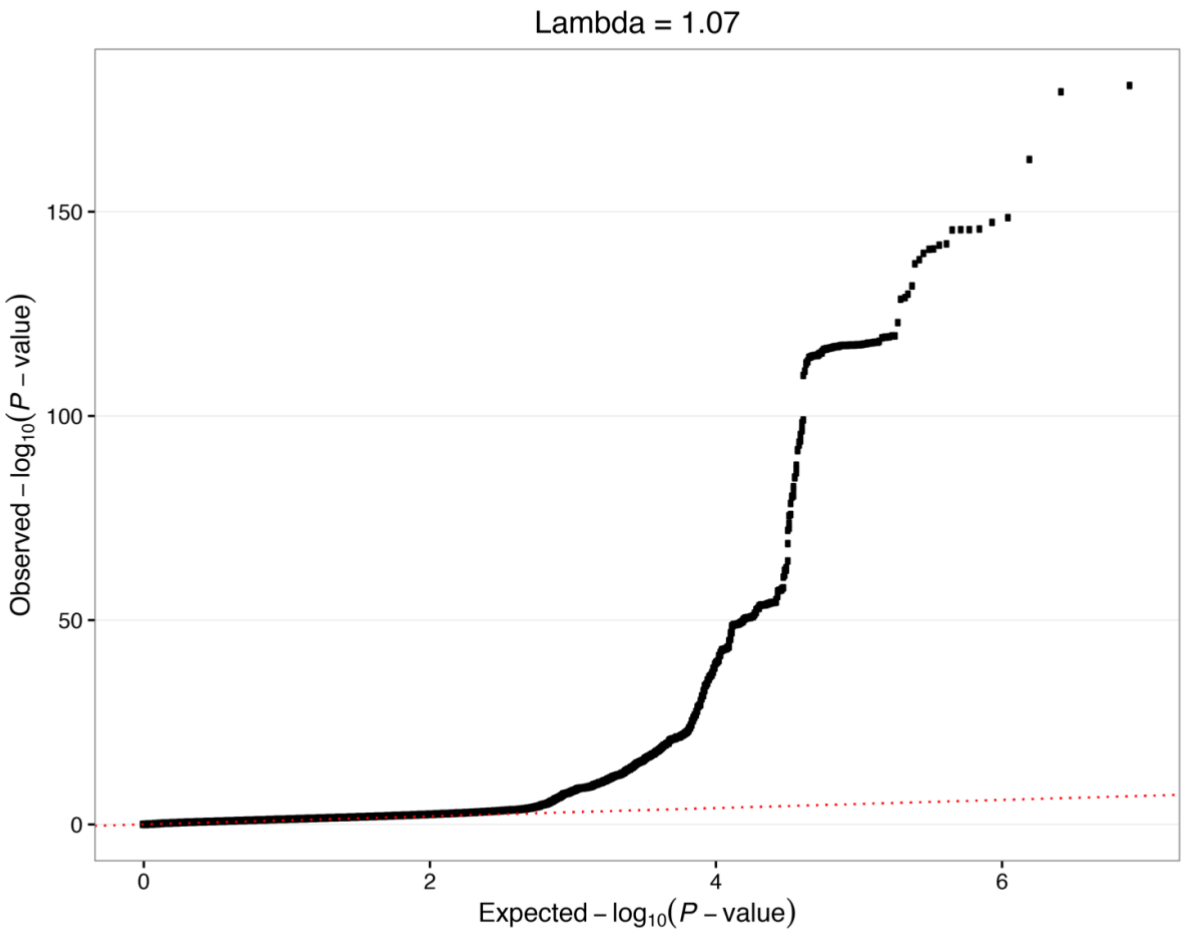

Supplementary Fig. 1

QQ-plot of all autosomal genetic variants tested for an effect on X-chromosomal methylation. Negative $\log_{10}$-transformed overall *P*-values (y-axis) are plotted against what is expected under the assumption of no effect. A clear deviation shows that several SNPs show an effect on X-chromosomal methylation. The value of lambda (1.07) suggests no residual confounding is present.

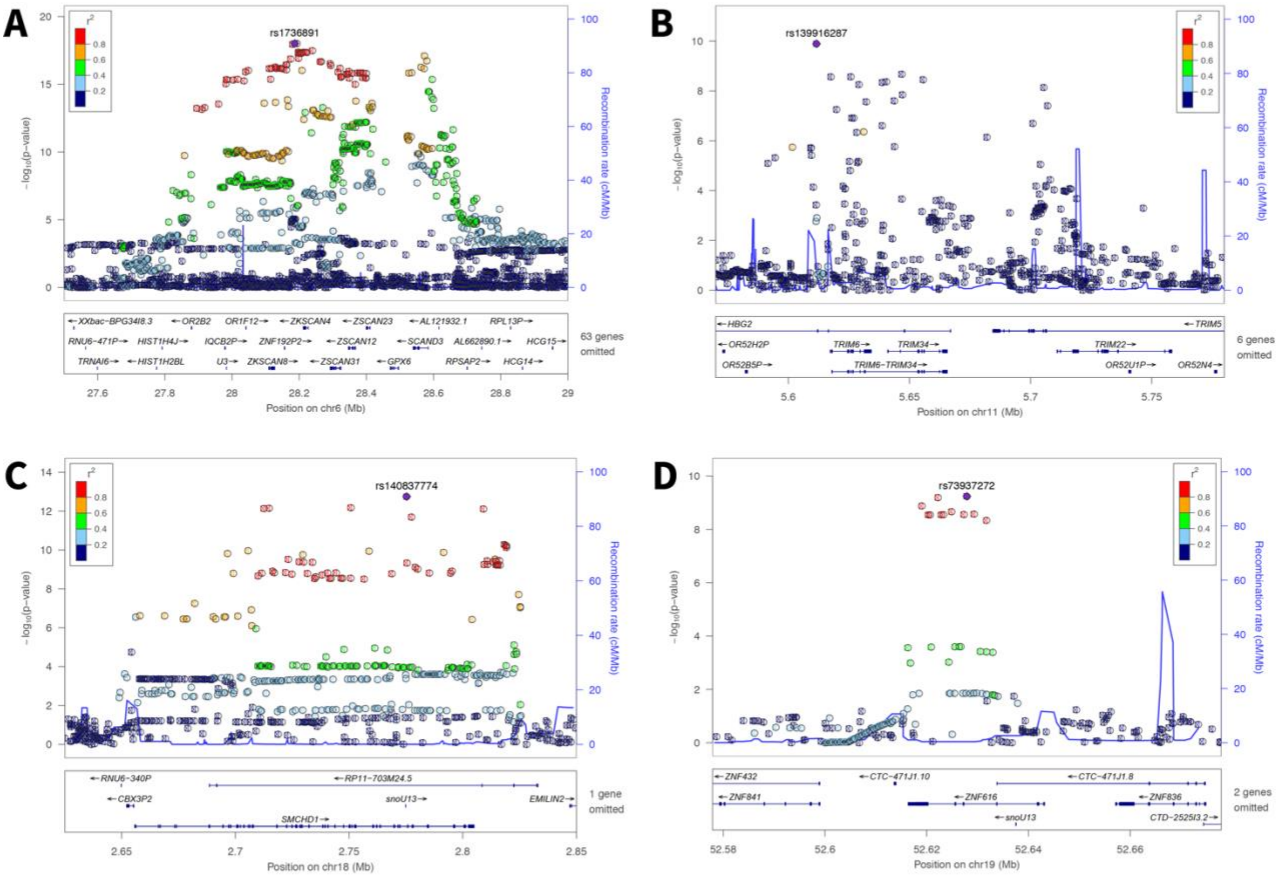

Supplementary Fig. 2

LocusZoom plots of the four autosomal loci having female-specific effects on overall X-chromosomal methylation. Depicted are the *ZSCAN9* (A), *TRIM6*/*HBG2* (B), *SMCHD1*/*METTL4* (C), and *ZNF616* (D) loci. Each dot represents the $\log_{10}$-transformed overall *P*-value of an association with methylation levels at any X-chromosomal CpG. Colors indicate the R^2 with the sentinel variant, which is signified by its rs-number.

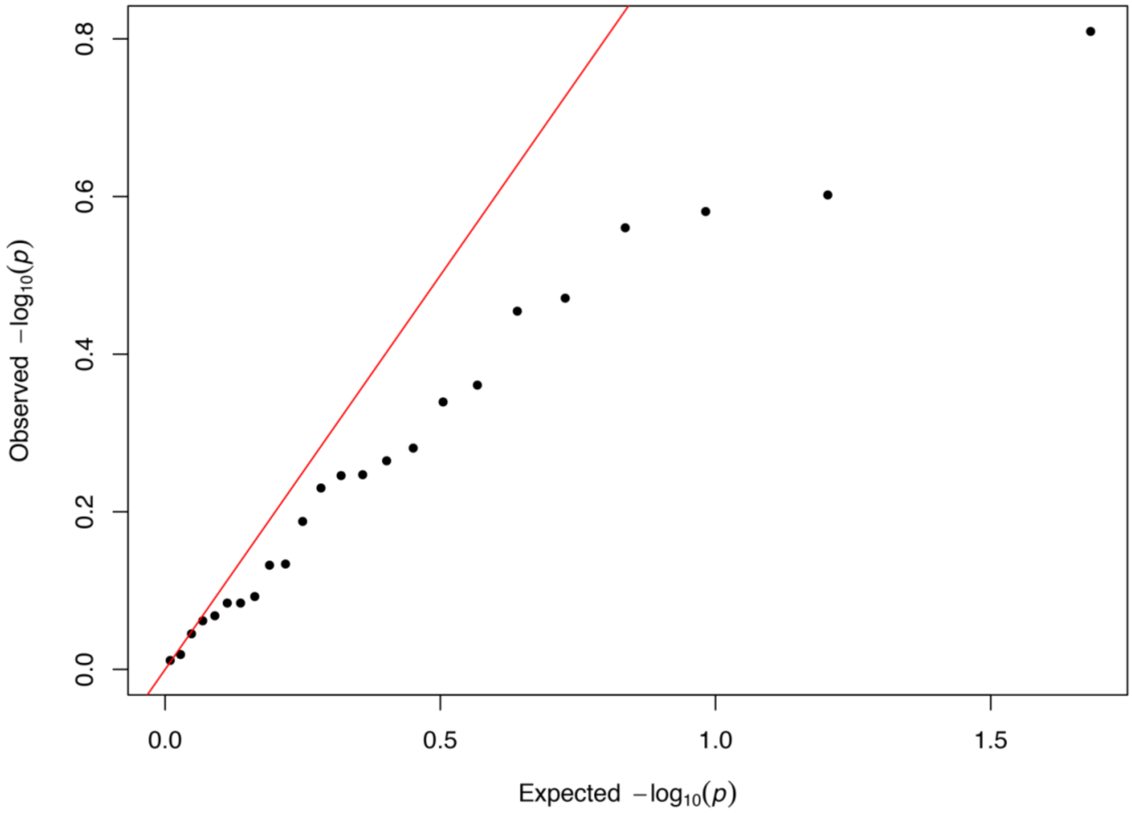

Supplementary Fig. 3

QQ-plot of the four sentinel variants tested for an effect on all of the measured cell counts. Tested cell types are neutrophils, monocytes, eosinophils, basophils, and red blood cell counts; lymphocytes were left out due to a strong correlation with neutrophils counts, corrected for age and known batch effects. Dots represent the $-\log_{10}$ transformed *P*-values. No clear upwards deviation from the diagonal (red, dotted line) indicates none of the variants have any effect on any of the cell counts.

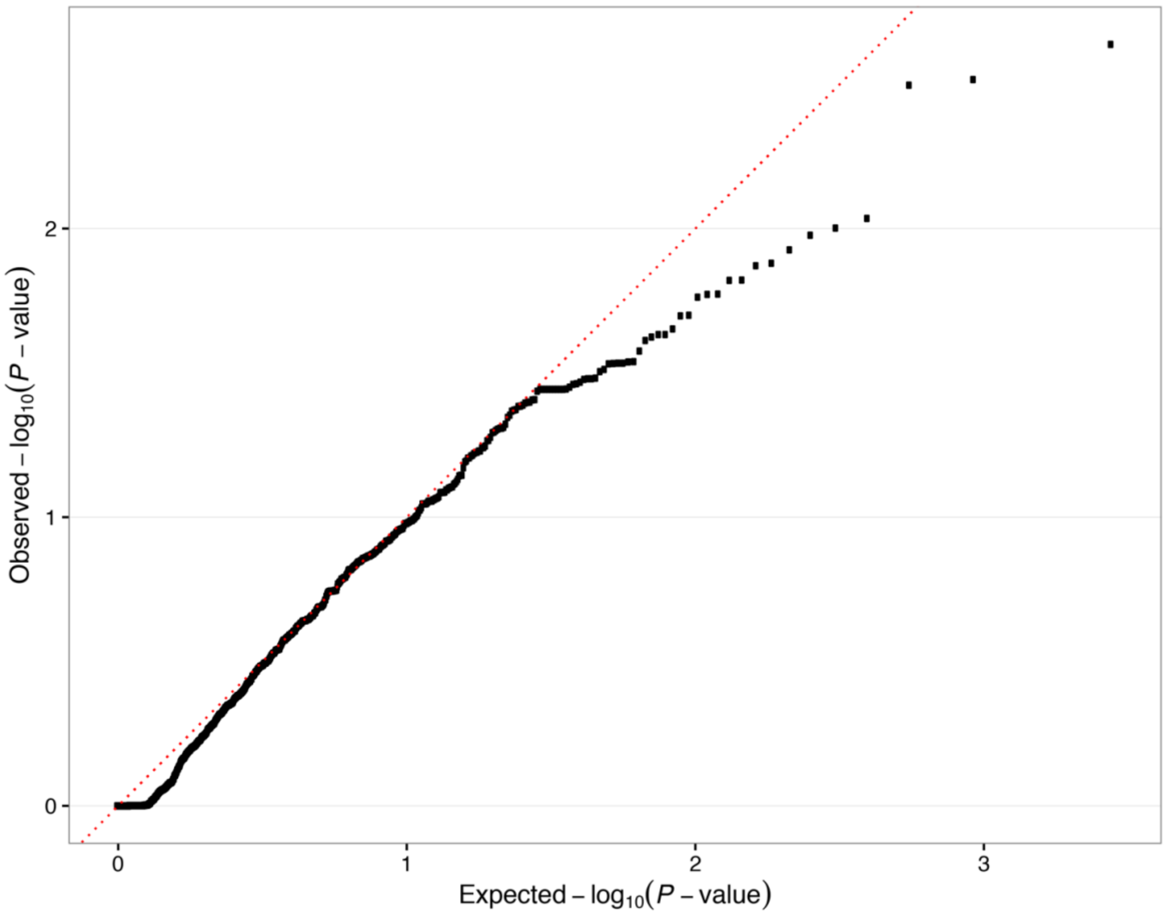

Supplementary Fig. 4

QQ-plot of all autosomal genetic variants known to affect blood composition[19,20] tested for an effect on X-chromosomal methylation in our data. Each dot represents the log_{10}-transformed overall *P*-value of any association with methylation levels at any X-chromosomal CpG. No clear deviation upwards of the diagonal line (in red, dotted) indicates there is no effect on blood composition.

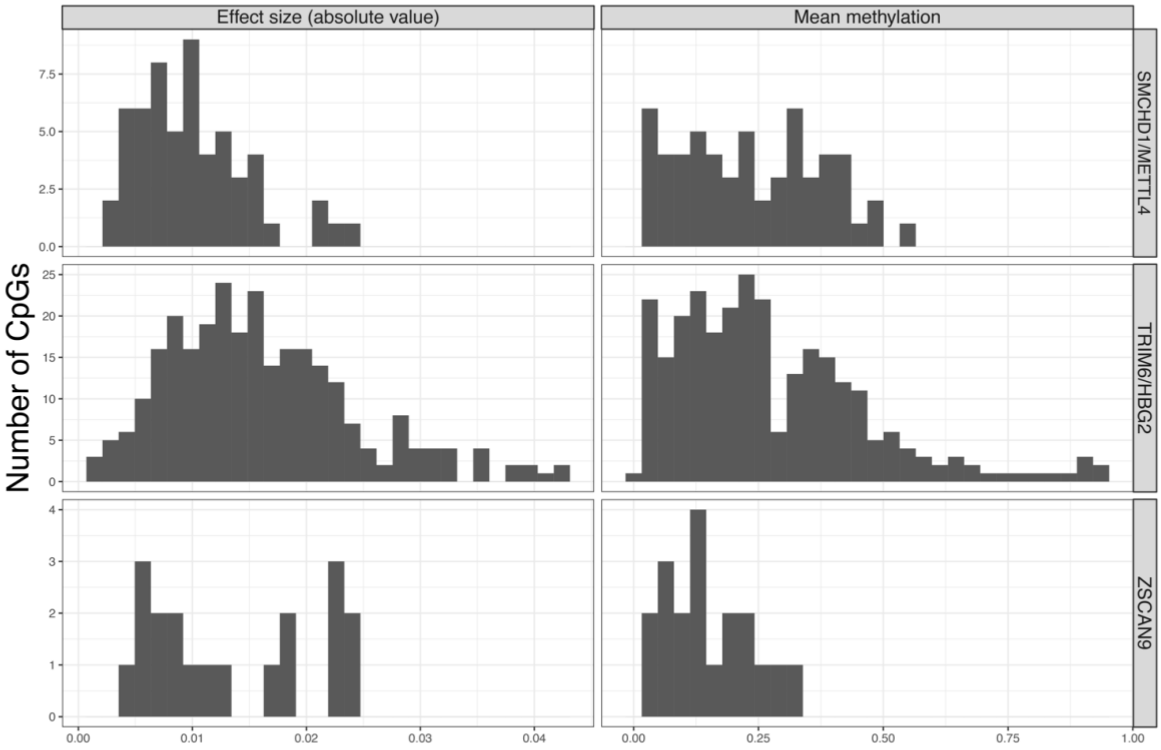

Supplementary Fig. 5

Distributions of the absolute values of the effect sizes and mean methylation per CpG for each of the autosomal loci. The distributions show small to modest effect sizes for mostly moderately methylation CpGs.

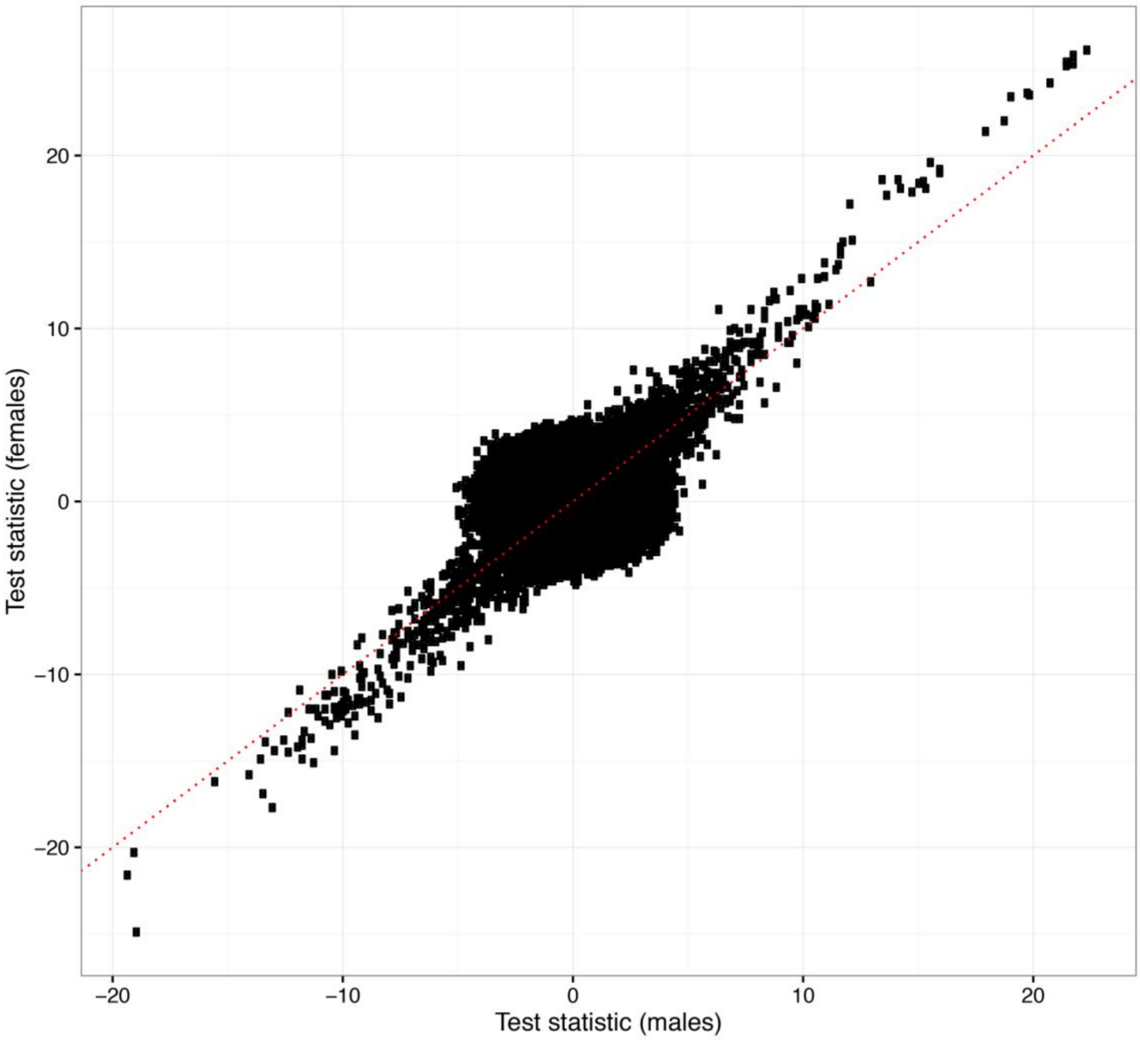

Supplementary Fig. 6

Test statistics showing the effect of all four sentinel variants on autosomal methylation *in trans* (> 5Mb). Each dot represents one tested association between any single sentinel variant and any single CpG. The x-axis shows the test-statistics as computed in male samples only (N_{males} = 1,398), whereas the y-axis shows the test-statistics in female samples only ($N_{females}$ = 1,867). These stratified analyses by sex show the effects of the four sentinel variants on autosomal CpGs are very similar between male and female samples. The slight deviation from the diagonal can be attributed to be the result from the different number of females and males.

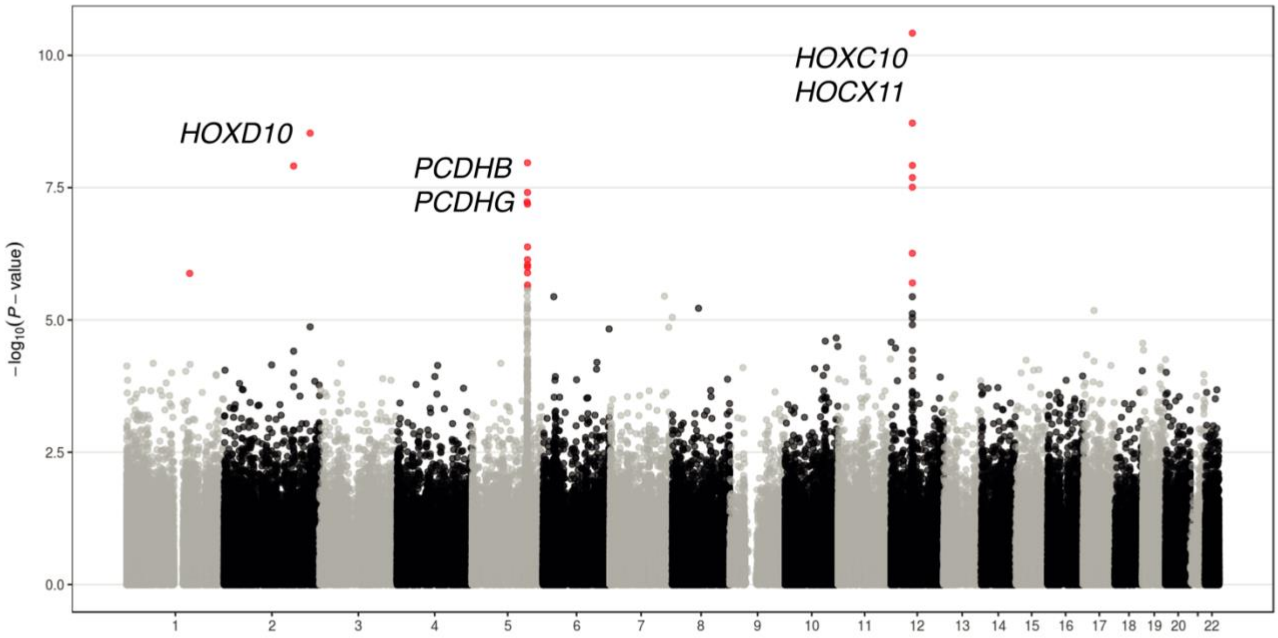

Supplementary Fig. 7

Manhattan plot for the *SMCHD1*/*METTL4* locus. The –$\log_{10}$(*P*-values) indicate the strength of the association with DNA methylation at autosomal CpGs *in trans* (>5Mb). The *SMCHD1*/*METTL4* locus associates with DNA methylation at 20 CpGs, located near several genes from the *HOX* and protocadherin clusters, known *SMCHD1* targets[25,32].

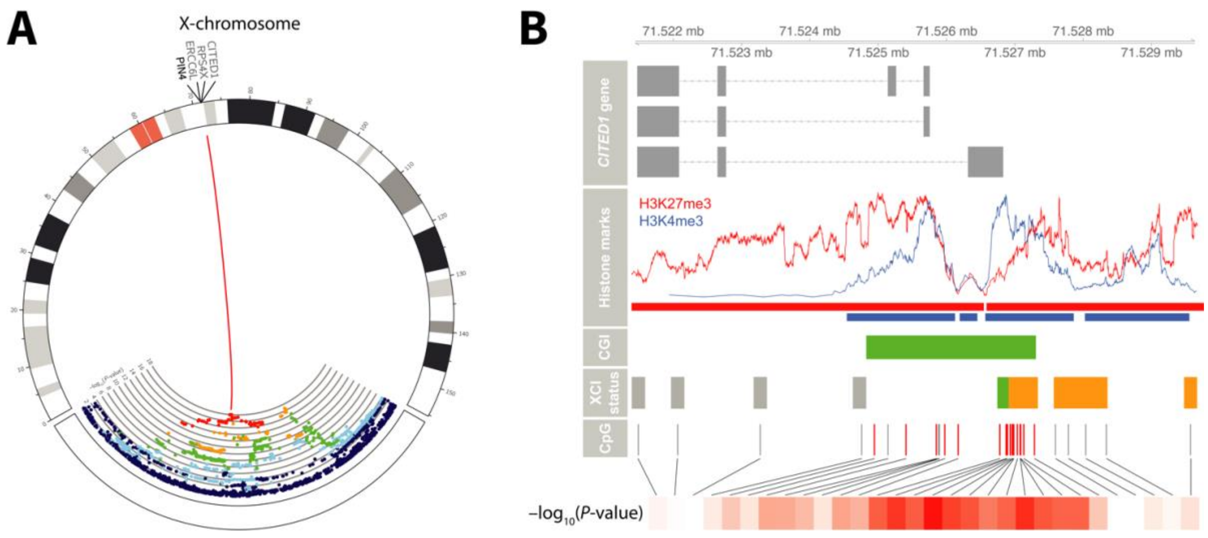

Supplementary Fig. 8

The *ZSCAN9* locus associates with DNA methylation at a single X-chromosomal CpG island near the *CITED1* gene. The *ZSCAN9* locus and its effects on X-chromosomal methylation are depicted. The colored dots indicate LD (red: $R^2 \geq 0.8$; orange: $0.6 \leq R^2 < 0.8$; green: $0.4 \leq R^2 < 0.6$; light blue: $0.2 \leq R^2 < 0.4$; dark blue: R^2 $0.6 \leq 0.2$), the y-axis shows the $-\log_{10}$(*P-value*) of the association with overall X-chromosomal methylation, and the line colors in the Circos plot indicate the direction of the effect (red: hypomethylation, blue: hypermethylation). **A)** The *ZSCAN9* locus shows a fair amount of LD, as indicated by the colored dots. The *ZSCAN9* locus strongly associates with *ZSCAN9* expression ($P = 2.5 \times 10^{-49}$) and 19 CpGs, all mapping to the same CpG island (mean effect size 1.3% per allele), where 17 CpGs (89.5%) are also associated with the *SMCHD1*/*METTL4* locus. The A-allele of rs1736891 is associated with reduced *ZSCAN9* expression and hypomethylation at all 19 CpGs associated with the *ZSCAN9* locus, where the changes in methylation are associated with *PIN4* expression (in bold). **B)** Depiction of the CpG island whose CpGs associate with the *ZSCAN9* locus. The enrichments of CpGs in certain genomic regions are similar to those found for the *SMCHD1*/*METTL4*

locus. Most importantly, the associated CpGs are also located in a region known to variably escape X-chromosome inactivation[10] (fourth row, orange bars).

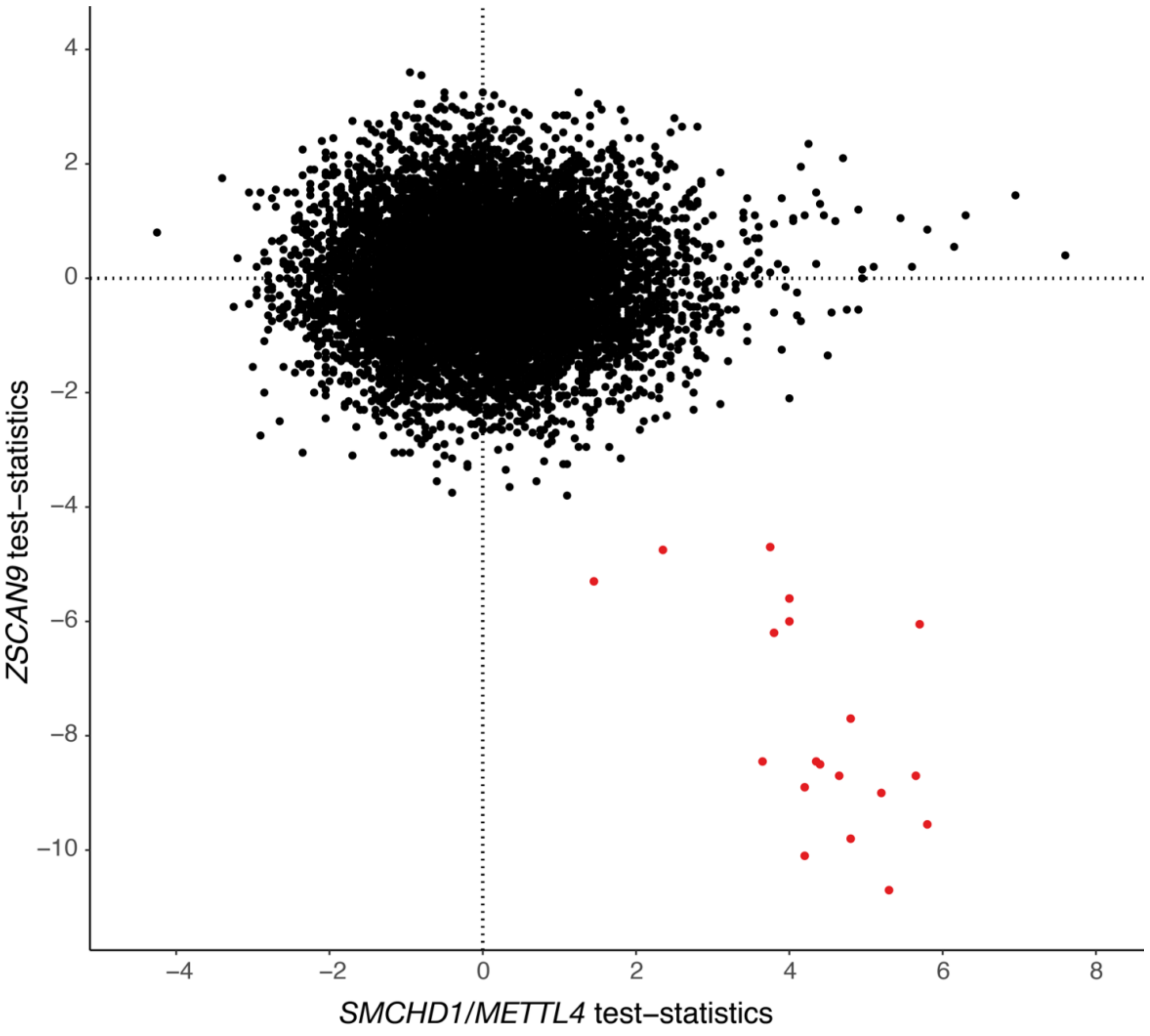

Supplementary Fig. 9

Test statistics for the *SMCHD1/METTL4* and *ZSCAN9* loci on DNA methylation at all X-chromosomal CpGs. Each dot represents one tested association between the two sentinel variants and any single X-chromosomal CpG. The red dots along the diagonal show test-statistics corresponding to CpGs in the CGI associated with genetic variation at both loci, indicating similar effects.
