# Supplementary material for: Autosomal genetic variation is associated with DNA methylation in regions variably escaping X-chromosome inactivation
Source: Nat Commun. 2018 Sep 14;9:3738. doi: 10.1038/s41467-018-05714-3 (PMC6138682; doi:10.1038/s41467-018-05714-3)
Supplement: Supplementary file 14 — Description of Additional Supplementary Files [file 41467_2018_5714_MOESM14_ESM.pdf]

Descriptions of Supplementary Data files

Supplementary Data 1
Number of female and male samples per cohort used in the discovery phase.

Supplementary Data 2
Characteristics of the four female-specific loci identified in the discovery cohort.

Supplementary Data 3
The 48 sentinel variants corresponding to the 48 loci identified in female samples, and tested for an effect in males. For each variant, the table associated *P*-values for females and males, the number of associated CpGs, the nearest gene, and mean effect sizes over the associated CpGs.

Supplementary Data 4
Test-statics for the association of three sentinel variants with X-chromosomal DNA methylation, both uncorrected and corrected for nearby X-chromosomal genetic variants.

Supplementary Data 5
All variants known to affect blood composition[19,20] and their associated *P*-values relating to the association with X-chromosomal DNA methylation. None of the variants reach statistical significance after correction for multiple testing, indicating these variants do not confound the analysis through blood composition.

Supplementary Data 6
Re-testing the four female-specific sentinel variants corrected for nearby (1Mb) variants known to affect blood composition[19,20], or without correcting for the latent factors.

Supplementary Data 7
Results from the replication analysis of the four sentinel variants in independent cohorts.

Supplementary Data 8
Results from *cis*-eQTL mapping, relating the sentinel variants of the three replicated, female-specific sentinel variants to nearby genes (<250Kb).

Supplementary Data 9
Results from *trans*-meQTL mapping of the three sentinel variants on all individual X-chromosomal CpGs.

Supplementary Data 10
X-chromosomal CpGs and the significant associations to nearby X-chromosomal genes (<250Kb).

Supplementary Data 11
Trans-meQTL mapping of the three sentinel variants on all autosomal CpGs *in trans* (>5Mb).
